# Neutrophils—Important Communicators in Systemic Lupus Erythematosus and Antiphospholipid Syndrome

**DOI:** 10.3389/fimmu.2019.02734

**Published:** 2019-11-22

**Authors:** Lina Wirestam, Sabine Arve, Petrus Linge, Anders A. Bengtsson

**Affiliations:** Section of Rheumatology, Department of Clinical Sciences Lund, Lund University, Lund, Sweden

**Keywords:** neutrophils, reactive oxygen species, neutrophil extracellular traps, systemic lupus erythematosus, antiphospholipid syndrome

## Abstract

Systemic lupus erythematosus (SLE) and antiphospholipid syndrome (APS) are two autoimmune diseases that can occur together or separately. Insights into the pathogenesis have revealed similarities, such as development of autoantibodies targeting subcellular antigens as well as a shared increased risk of cardiovascular morbidity, potentially due to mutual pathologic mechanisms. In this review, we will address the evidence implicating neutrophils in the pathogenesis of these conditions, highlighting their shared features. The neutrophil is the most abundant leukocyte, recognized for its role in infectious and inflammatory diseases, but dysregulation of neutrophil effector functions, including phagocytosis, oxidative burst and formation of neutrophil extracellular traps (NETs) may also contribute to an autoimmune process. The phenotype of neutrophils in SLE and APS differs from neutrophils of healthy individuals, where neutrophils in SLE and APS are activated and prone to aggregate. A specific subset of low-density neutrophils with different function compared to normal-density neutrophils can also be found within the peripheral blood mononuclear cell (PBMC) fraction after density gradient centrifugation of whole blood. Neutrophil phagocytosis is required for regular clearance of cell remnants and nuclear material. Reactive oxygen species (ROS) released by neutrophils during oxidative burst are important for immune suppression and impairment of ROS production is seen in SLE. NETs mediate pathology in both SLE and APS via several mechanisms, including exposure of autoantigens, priming of T-cells and activation of autoreactive B-cells. NETs are also involved in cardiovascular events by forming a pro-thrombotic scaffolding surface. Lastly, neutrophils communicate with other cells by producing cytokines, such as Interferon (IFN) -α, and via direct cell-cell contact. Physiological neutrophil effector functions are necessary to prevent autoimmunity, but in SLE and APS these are altered.

## Key Points

- The neutrophil effector functions; phagocytosis, oxidative burst and formation of NETs, are all important for a successive host defense against pathogens.
- In SLE, neutrophils are more activated, have a lower phagocytic capacity, a decreased production of NOX2 ROS, an increase in mitochondrial ROS, and are more prone to spontaneously release NETs.
- In APS, neutrophils have an activated phenotype with increased aggregation, mitochondrial dysfunction with increased mitochondrial ROS production, and display enhanced spontaneous NET release.
- Dysregulated neutrophil functions are shared pathogenetic mechanisms in both SLE and APS, contributing to loss of tolerance.

## Introduction

The neutrophil is the most abundant leukocyte and important in most aspects of the immune system. Long thought of as a non-specific cell at the front line of defense against infections, often causing tissue damage on its way, the neutrophil is becoming increasingly recognized as a more sophisticated cell of vital importance for immune homeostasis. The innate immune system is the first line of host defense against invading microorganisms. Neutrophils are phagocytes circulating in the blood awaiting to be recruited to sites of infection with a primary role in the clearance of extracellular pathogens (1). The homing to inflamed tissues is dependent on interaction with endothelium and several adhesion molecules, such as selectins and integrins, for successful extravasation and migration. Neutrophils have a wide range of receptors that recognize microbial and fungal structures as well as complement receptors that facilitate the phagocytosis of pathogens. After the engulfment, the phagosome fuses with neutrophil granules containing antimicrobial peptides and proteins. The increased oxygen consumption, called the oxidative burst, results in formation of toxic superoxide anions and reactive oxygen species (ROS) enabling activation of the antimicrobial peptides and proteins. The neutrophils may also defend against pathogens by releasing their granules and chromatin, so they form extracellular fibers (2). These neutrophil extracellular traps (NETs) degrade virulence factors and kill bacteria. An overall decrease in neutrophils are deleterious and neutropenic patients are at high risk for mortality from severe and recurrent infections (1).

**Figure 1:**
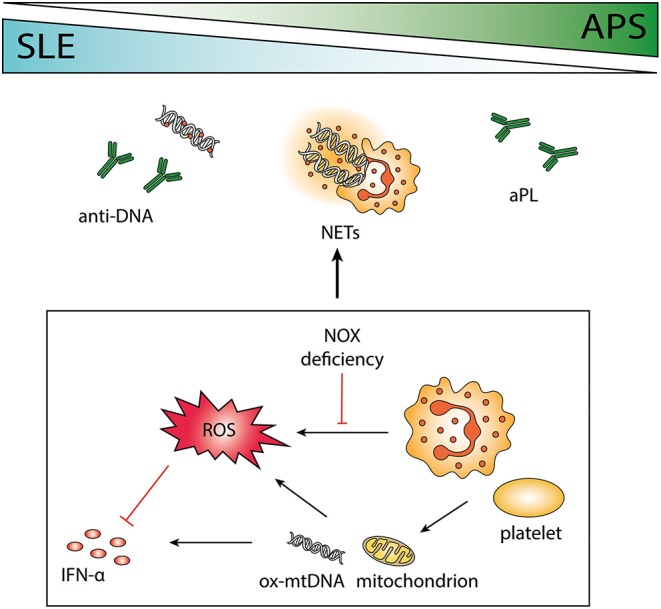
The role of neutrophils in systemic lupus erythematosus (SLE) and antiphospholipid syndrome (APS). SLE and APS are two autoimmune diseases that can occur together or separately in a patient with overlapping pathogenesis. Antibodies to phospholipids and DNA are characteristic of the two disorders, and the release of neutrophil extracellular traps (NETs) may contribute to the antigenic burden. Neutrophils are important immune regulators via the release of reactive oxygen species (ROS), e.g., by impeding interferon (IFN) -α. Polymorphisms causing deficient ROS production by NOX is associated with autoimmunity, and absence of functional NOX may force cells to use mitochondrial ROS instead. Both neutrophils and platelets can release mitochondria and mitochondrial DNA (mtDNA), highly potent in inducing IFN-α production. Neutrophil-platelet interactions increase ROS production and facilitate NET release.

The neutrophil effector functions; phagocytosis, oxidative burst and formation of NETs, are all renowned for their importance in host defense, but abnormalities in these functions are also associated with development of autoimmune disease. The specific mechanisms connecting each neutrophil effector function with autoimmune reactions in the context of systemic lupus erythematosus (SLE) and antiphospholipid syndrome (APS) will be discussed in detail in this review. The influence of neutrophils on the pathogenesis of SLE is more explored, less is known about the potential effect on the closely related pathogenesis of APS. In this review we will highlight unifying factors and by utilizing knowledge from studies in SLE, we anticipate a better understanding also of neutrophil impact on APS pathogenesis. See Figure 1 for a graphical overview of neutrophil contribution to the pathogenesis of both conditions.

## Systemic Lupus Erythematosus and Antiphospholipid Syndrome

SLE is a heterogenous inflammatory autoimmune disease with increased mortality and low quality of life, involving most organ systems, including joints, skin, kidneys and heart (3). Despite best available treatment, flares and disease activity are seen in most patients over time and the chronic inflammation contributes to the development of irreversible organ damage. Cardiovascular disease (CVD) represents the most common cause of morbidity and mortality (4). SLE mostly affects women in childbearing age and the prevalence in e.g., Sweden is estimated to 65 in 100,000 (5). APS is an autoimmune disease characterized by thrombosis and pregnancy related complications such as miscarriage and preeclampsia, in the presence of antiphospholipid antibodies (aPL). An APS diagnosis is considered if at least one of the clinical criteria (i.e., thrombosis or pregnancy morbidity) and one of the laboratory criteria (i.e., persistently positive lupus anticoagulant test and/or presence of anti-cardiolipin or anti-β2-glycoprotein-I antibodies at moderate to high titer) are met (6). In addition to the typical manifestations of thrombosis, there are other “non-criteria” manifestations (7). Stagnation of blood flow can lead to livedo reticularis, a red or bluish net-like discoloration of the skin. Heart valve abnormalities, such as thickening of the heart valve occur in up to a third of all APS patients. The clinical spectrum also includes thrombocytopenia, cognitive disorder, seizures, and renal vasculopathy, resembling lupus and similar to lupus APS may have the features of a multiorgan systemic disease. The prevalence is 40–50 in 100,000 and in about half of the cases APS occurs as a primary condition, whereas in the remaining half, it occurs secondary to another autoimmune disease, most notably SLE (8, 9). APS shares both pathogenetic and clinical features with SLE. One or more of the major aPL or lupus anticoagulans (LA) are found in ~20–30% of SLE patients (10, 11) and aPL are more frequent in first-degree relatives of SLE or primary APS patients, suggesting a genetic susceptibility (12). Familial clustering of primary APS and SLE associated APS has been observed and identified predisposing genetic factors include HLA variants (primarily DR4 and DR7) (13, 14). There are conflicting data regarding non-HLA gene association with APS, including IRF5 and STAT4 (15–17). Thus, the two diseases share genetic predisposition and clinical manifestations, suggestive of shared pathogenetic mechanisms.

Inflammation plays a central role in the development of CVD and patients with chronic inflammatory diseases in general are predisposed. SLE patients have a 2–10-fold increased risk of developing CVD, such as stroke and myocardial infarction, compared to the general population (18), and premenopausal women have the greatest fold difference with a 50-fold increased risk (19). Traditional risk factors such as smoking, dyslipidemia and hypertension are associated with atherosclerosis, but cannot fully explain the increased risk seen in SLE patients. Inflammatory processes driving the SLE pathogenesis also contributes to the development of CVD. An activated type I interferon (IFN) system has effects on the endothelium contributing to endothelial dysfunction, an early step in the development of atherosclerosis (20). The underlying mechanisms of how presence of aPL can result in clinical manifestations are still not fully understood, but alterations of the endothelia, formation of aPL immune complexes, activation of platelets and the complement system have all been suggested to contribute to the pro-coagulative state causing clinical manifestations (7). SLE patients, also without APS, have a 2–5-fold increased risk of venous thrombosis (21), and an even higher risk is seen in patients positive for aPL (11). The interplay between SLE and APS is complex and immune dysregulation appears to play a key role in driving both of the pathogeneses and contributing to CVD and venous thrombosis.

Both SLE and APS are characterized by antibodies directed against self-antigens, normally found within cells, but exposed during cell death. In SLE, apoptotic cells that are not sufficiently eliminated may undergo secondary necrosis, thereby releasing autoantigens (e.g., nucleotide containing structures) and endogenous danger signals that promote inflammation (22). Furthermore, autoantibodies and nucleic acid containing antigens form immune complexes (ICs) which can induce production of IFN-α by plasmacytoid dendritic cells (pDCs) via Toll-like receptor (TLR) -7 or -9 (23). Neutrophils may also produce IFN-α in response to chromatin under certain circumstances (24). Binding of IFN-α to its receptor initiates the Janus kinase (JAK)/signal transducer and activator of transcription (STAT) pathway with activation of IFN regulated genes (25). The majority of patients with SLE express increased levels of IFN-inducible genes, i.e., “a type I IFN signature,” and/or have raised circulating levels of IFN-α (26, 27). The role of type I IFN in SLE is well established, however, there is evidence of an active type I IFN system also in APS. An IFN signature is found in primary APS and associates with endothelial progenitor dysfunction (28). Similar to SLE, development of transient features of APS may evolve during IFN-α treatment. The development of low titers of anti-cardiolipin antibodies during interferon alpha therapy in chronic hepatitis C has been reported (29), and APS may evolve during PEG IFN-α therapy (30). The possible importance of IFN-α in APS pathogenesis is illustrated by the fact that aPLs may also contribute to IFN-α production, by inducing translocation of TLR7 to the endosomes in pDCs, thereby priming them to internalize RNA (31). Of relevance for both SLE and APS, IFN-α is linked to increased activation of antigen presenting cells, augmented antibody production, and increased apoptosis (26). Deficient physiologic clearance of circulating ICs can lead to IC-deposition in tissues and cause inflammation. In this way, a vicious circle of increased apoptosis, impaired clearance, autoantigen exposure, autoantibody production, chronic inflammation, and tissue damage may proceed.

## Neutrophils and their Effector Functions in SLE and APS

### Neutrophil Phenotypes

Despite neutrophils being short lived and terminally differentiated cells, heterogeneity among neutrophil phenotypes, both regarding function and expression of surface markers, exist. In the circulation, there are naturally occurring differences between neutrophils depending on their age. Aged neutrophils will express less of adhesion molecule L-selectin and more of activation marker CD11b (32). In tissues, neutrophils have certain ability to polarize depending on the tissue specific milieu and in similarity with monocytes and macrophages neutrophils might polarize into a pro- [tumor necrosis factor (TNF) α-driven] or anti-inflammatory [transforming growth factor (TGF) β-driven] phenotype (32).

In SLE, neutrophils display an activated phenotype with increased aggregation and platelet-neutrophil complex formation compared to neutrophils of healthy controls, and SLE neutrophils are more prone to undergo apoptosis (33–36). Increased neutrophil activation is also seen in APS, and neutrophils from APS patients have an increased expression of cell adhesion genes and proteins resulting in increased neutrophil adhesiveness (37, 38). In models of fetal injury, aPLs generates complement protein C5a via the classical pathway, and C5a is a potent chemotactic factor and activator of neutrophils. C5a-mediated neutrophil infiltration is observed at sites of fetal resorption and depletion of neutrophils protects mice from aPL-mediated fetal injury (39).

A specific subset of granulocytes with low density and different properties compared to normal density granulocytes have been identified in several chronic inflammatory conditions, first discovered in SLE in 1986 (40). These neutrophil-like cells are found within the peripheral blood mononuclear cell (PBMC) fraction after density gradient centrifugation of whole blood, separated from normal neutrophils present in a higher density fraction. Two types of low-density neutrophils have been described in SLE; low-density granulocytes (LDGs) associated with a proinflammatory phenotype and neutrophil-like myeloid derived suppressor cells (PMN-MDSC) which have an anti-inflammatory phenotype (41, 42). These cell types share several features including density, morphology and CD-marker expression but differ markedly in their role in inflammation (Table 1). There are currently no consensus regarding whether these cells belong to the same or different cell types.

**Table 1:** Phenotypes and functions of neutrophils, LDG and PMN-MDSC.

	**LDG**	**PMN-MDSC**	**Neutrophil**	**References**
Density	Low (PBMC-fraction)	Low (PBMC-fraction)	Normal (PMN-fraction)	(43)
CD-markers	CD15^+^CD14^−^ CD10^+^CD14^−^ CD10^+^CD15^+^CD14^−^	CD11b^+^CD14^−^CD15^+^ CD11b^+^CD14^−^CD66^+^ CD11b^+^Gr-1^+^ CD15^+^LOX1^+^	CD11b^+^CD14^low^ CD15^+^CD16^+^ CD62L^+^	(43–47)
Morphology	Neutrophil-like Less segmented nucleus	Neutrophil-like Less segmented nucleus	Neutrophil Segmented nucleus	(27, 44)
ROS	+	+++	++	(43, 48)
NETs	+++	+	++	(49, 50)
Phagocytosis	+	?	++	(43)
Immune suppression	-	++	+	(44, 51, 52)
Cytokine production	IFN-α, TNFα, IL-8, IL-6	IL-10		(43, 53)
Gene expression	Granule enzymes Cytokines	Granule enzymes Cell cycle-related proteins		(27, 49, 54)

LDGs are characterized by proinflammatory features such as production of cytokines and spontaneous release of NETs containing oxidized mitochondrial DNA (43, 44, 49, 55). Compared to normal neutrophils, LDGs have impaired oxidative burst and phagocytosis, but an enhanced ability for NET release and cytokine production (43, 48). Proinflammatory cytokines produced by LDGs include type I IFN, IFN γ, IL-6, IL-8 and TNFα, all of importance in SLE pathogenesis (43). NETs released from LDGs induce endothelial damage by activation of endothelial matrix metalloproteinase-2 via matrix metalloproteinase-9 present in NETs (31). Moreover, LDG NETs contain enzymes such as myeloperoxidase and nitric oxide synthase which oxidize high density lipoprotein, making it proatherogenic (56, 57). In SLE, LDGs are associated with vascular damage (43, 58) and with disease activity in juvenile lupus (59). In APS, LDGs are enriched especially in patients with high titers of anti-β2-glycoprotein-I (60), antibodies capable of inducing NETosis (61, 62).

An increased NET release by LDGs may contribute to the high cardiovascular morbidity in both SLE and APS, and the importance of NETs will be discussed further in this review.

First described in cancer, MDSCs are defined as myeloid progenitor cells with suppressive effects on T-cells (51) and can be divided into two groups, monocyte-like (M-MDSC) and neutrophil-like (PMN-MDSC), both subtypes being immunosuppressive. PMN-MDSC exert their immunosuppressive effects mainly via the production of ROS (52, 63). In murine models of SLE, PMN-MDSCs have been demonstrated to induce expansion of regulatory B- and T-cells, decrease T-cell activation, suppress B-cell differentiation and autoantibody production, as well as ameliorate SLE symptoms (50, 53, 64, 65). Despite several studies on PMN-MDSCs in murine autoimmunity, they have not been characterized in human disease. Two studies investigating MDSCs in SLE patients demonstrate that levels of cells with PMN-MDSC phenotype correlate with increased disease activity (66), and interferon signature (67), but without suppressing T-cell proliferation or activation, thus being LDGs rather than MDSCs. To our knowledge no work regarding MDSCs in APS is published. Clearly, MDSCs in the context of APS and SLE needs further attention to scrutinize their role in humans.

### Neutrophil Phagocytosis and Clearance

Clearance deficiency of dying cells is involved in the etiology of autoimmunity and there is an observed increase of apoptotic neutrophils in combination with an impaired phagocytosis by macrophages in SLE (36, 68). In the absence of a proper clearance, apoptotic cells may turn into secondary necrotic cells (SNECs), releasing autoantigens and danger signals (22). The first neutrophil abnormality described in SLE was the discovery of the so called LE-cell (lupus erythematosus cell) first reported in 1948 in the bone marrow of SLE patients (69). The LE-cell is a blood granulocyte in which the nucleus after excessive phagocytosis of opsonized apoptotic cell remnants, closely resembling SNEC, become outstretched and pushed toward the edges of the cell (70, 71). A combination of antibodies to several different histone proteins promotes this phenomenon, increasing the uptake of nuclear material (72, 73).

Nuclear remnants in the circulation of healthy individuals are not phagocytosed, but rapidly degraded by DNases and C1q via the reticuloendothelial system. In SLE, impaired DNase activity or deficiency of complement proteins is common. Nuclear material, opsonized by antinuclear antibodies (ANA) and complement, is instead dependent on removal by phagocytosis by e.g., neutrophils (74), and autoantibodies recognizing SNECs promote neutrophilic phagocytosis (75).

Apoptotic cells which are not cleared become decorated with β2GPI and cardiolipin is reportedly translocated from the inner mitochondrial membrane to the cell surface during apoptosis, exposing targets for APS-related antibodies (76–78). Opsonization of apoptotic bodies may shift the clearance toward proinflammatory pathways. Thus, the reduced removal of apoptotic cell remnants may be an explanation to why SLE and APS are syndromes often occurring simultaneously and an indication of shared pathogenic mechanisms.

### Neutrophil Reactive Oxygen Species

**Figure 2:**
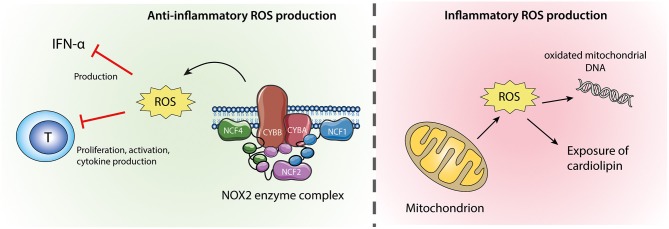
Neutrophil ROS from NOX2 and mitochondria. ROS produced by the NOX2 enzyme complex **(left)** are anti-inflammatory via their inhibitory effects on IFN-α production and T-cell activation. Mitochondrial ROS **(right)** might cause oxidation of mitochondrial DNA which if released from the cell is highly inflammatory. Excessive mitochondrial ROS and apoptosis can also result in translocation of cardiolipin from the inner to the outer mitochondrial membrane and the plasma membrane.

There are two sources of ROS in neutrophils (Figure 2). The most important source of ROS in neutrophils is generated from the NADPH oxidase 2 (NOX2) enzyme complex, which is selectively expressed in phagocytic cells and produces large amounts of ROS during oxidative burst. In addition, as in other cell types, neutrophil ROS can be formed by oxidative phosphorylation during mitochondrial ATP production (79). The role of ROS in autoimmune diseases is highly complex, where a normal production of ROS is necessary both for host defense during infection and redox regulation of the immune system. ROS may be toxic for host cells and have therefore traditionally been considered harmful, but the last decade of research have clearly demonstrated that NOX2-derived ROS are important second messengers and immune regulators, suppressing excessive inflammation (79).

#### NOX2-Derived ROS

The process of oxidative burst is highly controlled and only occurs in primed or stimulated cells, as the multicomponent NOX2 complex in resting cells is inactive, separated between the plasma membrane and the cytosol. The membrane proteins NOX2 and cytochrome b-245 alpha polypeptide (CYBA) form the catalytic flavocytochrome b_558_, using NADPH to produce superoxide (80). However, the production of reactive oxygen is only possible if the membrane proteins are associated with the cytosolic components neutrophil cytosolic factor 1 (NCF1), NCF2 and a small GTPase (i.e., Rac 1 or 2). Upon neutrophil activation, NCF1 is heavily phosphorylated by protein kinase C and the subsequent conformational change enables binding to NCF2 and NCF4. The cytosolic complex is transported to the plasma membrane where it docks to flavocytochrome b_558_ resulting in activation of the NOX2 complex (80, 81).

Chronic granulomatous disease (CGD) is a primary immunodeficiency caused by mutations in the NOX2 subunits (82), characterized by recurrent severe infections and lupus-like features due to impaired ROS production via NOX2. Genetic variations in several NOX2 complex components have been associated with multiple autoimmune diseases (83–87), highlighting the importance of a functional NOX2 complex for maintenance of a healthy immune system. Several independent studies have found a connection between impaired ROS production and SLE (83–85, 88), and in animal models of SLE deficiency of NOX2 function results in excacerbated disease (89, 90). The NCF1-339 single nucleotide polymorphism (SNP), where the minor allele T reduces the burst capacity of neutrophils, is one of the strongest identified genetic associations with SLE with an OR of 3.7 and an allele frequency of 17% (83). The NCF1-339 T allele has been shown to increase the expression of type I IFN regulated genes and associate with a younger age at SLE diagnosis. Genetic variants in NCF2 and NCF4 generating decreased ROS production have also been associated with SLE and/or other rheumatic diseases (85, 87).

In NOX2 expressing cells such as neutrophils, ROS produced by the NOX2 complex are important for regulation of autophagy (91), clearance associated LC3 (microtubule-associated protein 1 light chain 3α)-mediated phagocytosis (92), pH-regulation in endosomes and phagosomes for quiescent handling of necrotic material (73, 93), as well as degranulation (94) and release of NETs (95, 96). Perhaps even more important are the use of ROS as a messenger molecule. NOX2 derived ROS have suppressive effects on CD4+ and CD8+ T-cell proliferation and pro-inflammatory cytokine production, which is abrogated in the presence of a ROS scavenger (63, 97). When released within the immunological synapse, ROS can downregulate T-cell reactivity by inhibiting T-cell receptor activation (98). Additionally, although not investigated in neutrophils, macrophages can induce the expansion of regulatory T-cells in a NOX2 dependent manner (99). This may be relevant since regulatory T-cells are important for maintaining tolerance, and the number of cells and the function is reduced in SLE (100), while studies in APS show contradictory results (101, 102). Of particular importance in SLE is the inhibitory effect ROS has on the production of IFN (103, 104). Interestingly, neutrophils from APS patients demonstrate a proinflammatory signature with overexpression of IFN signaling genes (37, 105) and both mice and CGD patients, which lack NOX2 activity, display an IFN signature and upregulation of STAT1 (106). Deficiency of ROS may oppose tolerance and increase IFN-α, potent in driving the SLE pathogenesis and possibly also APS.

Little is known about how NOX2-derived ROS are related to APS. Oxidative stress unfolds the ring conformation of β2GPI and increases the immunogenicity by exposing domain I, an epitope for pathogenic autoantibodies (107). The activation of NOX2 by aPL in monocytes and dendritic cells upregulates TLR-7 and−8 with subsequent proinflammatory cytokine production (108). Moreover, aPL activation of NOX2 also results in induction of tissue factor. Some studies suggest that high, rather than low, NOX2-derived ROS are contributing to APS (109). More research is needed to clarify the role of ROS in APS.

#### Mitochondrial ROS

In absence of a functional NOX2 complex, neutrophils may become more reliant on mitochondrial ROS. Proinflammatory LDGs have weak oxidative burst but enhanced mitochondrial ROS production and release mitochondria-derived NETs (55). The shifted balance from NOX2 to mitochondrial ROS is probably of importance for the proinflammatory phenotype of LDG. Enhanced production of mitochondrial ROS cause oxidation of the unprotected mitochondrial DNA. In SLE neutrophils, oxidized mitochondrial DNA is not properly disposed of and the neutrophils instead extrude oxidized mitochondrial DNA with potent IFN-stimulatory effect on pDC (110). Additionally, inhibition of mitochondrial ROS in a lupus mouse model reduces disease severity and type I IFN response (55).

In APS, mitochondrial ROS may be of pathogenetic importance. Healthy monocytes exposed to aPL leads to mitochondrial dysfunction and inhibition of mitochondrial ROS reduces the expression of prothrombotic and proinflammatory markers (111). Cardiolipin is exclusively expressed by mitochondria and becomes exposed on the outer membrane upon mitochondrial dysfunction (112). Hence, decreased NOX2 activity, increased mitochondrial ROS production and mitochondrial dysfunction may expose cardiolipin, hypothetically leading to development of APS. Mitochondria decorated in NETs or as a consequence of impaired mitophagy (55, 110) are important for autoimmune reactions toward this antigen, and anti-mitochondrial antibodies are detected in both SLE and APS (113, 114). Thus, an altered mitochondrial function may drive the disease toward APS.

### Neutrophil Extracellular Traps

**Figure 3:**
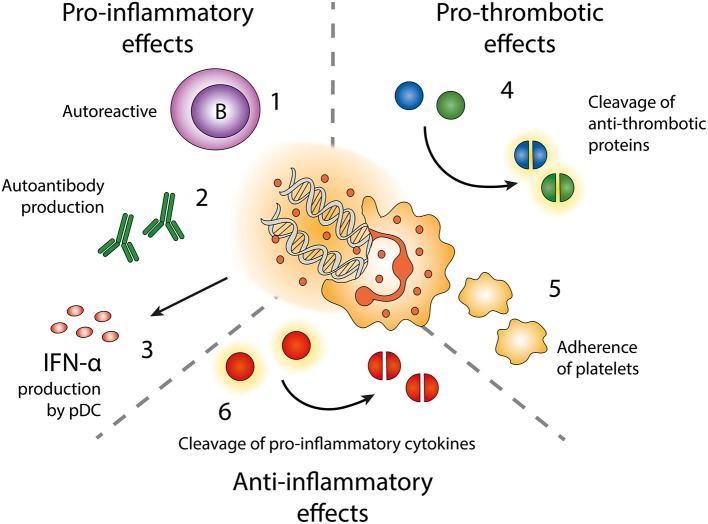
Effects of neutrophil extracellular traps (NETs). Exposure of autoantigens leads to activation of autoreactive B-cells (1) and autoantibody production (2). NETs and NET-containing IC activate pDC to produce IFN-α (3). NETs contain enzymes cleaving anti-thrombotic proteins (4) resulting in activation of the coagulation cascade. NETs also form a scaffold onto which platelets can adhere and form thrombi (5). NET-related enzymes can also cleave and inactivate pro-inflammatory cytokines (6).

NETs were first described in host response via the ability to capture and kill bacteria (2), but has later been found to be implicated in a myriad of different conditions ranging from coagulation to cancer to autoimmunity (115, 116). We now know that the formation of NETs is equally important as the other more studied neutrophil effector functions in the immune system (Figure 3).

NETs are released from neutrophils in response to a wide variety of stimuli such as microbes and pathogen associated molecular patterns (PAMPs), but also sterile stimuli including cytokines, antibodies, immune complexes and chemicals such as protein kinase C activator phorbol-myristate-acetate (PMA) and calcium ionophores (2, 95, 96, 117–119). Several intracellular NET-inducing signaling pathways have been described (120), most of which include either generation of superoxide via the NOX2 complex or citrullination of proteins by PAD4 (95, 121), and in some instances by mitochondria derived ROS (55, 119). The process of NET release is often accompanied by neutrophil cell death (NETosis), but “vital NETosis” where the neutrophil remain viable after NET release is also described (122, 123). Depending on stimulus and intracellular pathway involved, the content of released NETs varies. DNA in NETs might origin either from the nucleus, the mitochondria or both (2, 55, 124, 125), and proteins may vary both in quantity and presence of post-translational-modifications such as citrullination (126, 127). NETs seem to have different immunogenic effects depending on their composition. Integration of multiple signals, such as environmental triggers, metabolic state and signals from the tethered phagocytic cargo, determine the decision of neutrophils to phagocytose or generate NETs (128). Defects in phagocytosis may lead to intravascular generation of NETs, promoting vascular inflammation seen in diseases characterized by defective clearance.

NETs expose epitopes normally shielded by the plasma membrane, which if not sufficiently cleared contributes to the autoantigenic burden, as demonstrated in several diseases including SLE, RA and small-vessel vasculitis (129–132). Enhanced NET release and impaired clearance of NET components are seen in both SLE and APS (62, 131, 133, 134). In SLE there is more and more evidence of involvement of NETs, as neutrophils of patients with SLE are more prone to spontaneously release NETs (33, 49), NET-remnants can be found in the circulation (135) and depositions in skin and kidney glomeruli (49). The NETs have several downstream effects on other cell types, such as priming of T-cells by reducing their activation threshold and activation of autoreactive B-cells (136, 137). NET-derived immune complexes trigger polyclonal B cell activation via TLR9, but also expand self-reactive memory B cells (136). Moreover, immune complexes formed by autoantibodies and NET-components can activate pDCs, resulting in production of IFN-α (55, 117, 125). Thus, NETs do not only contribute to SLE by antigen exposure but also by its impact on other immune cells.

Similarly, patients with primary APS also display enhanced spontaneous NET release (62). Plasma of patients with APS (both primary and secondary) as well as aPL alone can induce NETs from healthy neutrophils (61, 138), and NET release correlates with circulating levels of aPL (62). Deposition of NETs can be found in intervillous tissue of pre-eclampsia pregnancies (139), possibly contributing to the pregnancy morbidities present in APS. In a study by Meng et al. mice treated with IgG isolated from APS patients demonstrated exaggerated NET formation and thrombosis, where NETs were found within the thrombi (140).

In the vasculature, NETs can form a scaffolding structure onto which platelets aggregate and form thrombi (141, 142). Activated platelets release the damage-associated molecular pattern high-mobility group box protein 1 (HMGB1) to neutrophils, committing them to NET generation (142). Neutrophils stimulated with antiphospholipid antibodies release NETs and promote thrombosis (140). NETs are also found in both arterial and venous thrombosis without underlying autoimmune disease [reviewed in Laridan et al. (143)]. NETs can bind factor XII and cooperate with platelets to activate the intrinsic pathway (144). Neutrophil proteases elastase and cathepsin G present in NETs cleave the anti-coagulant proteins antithrombin III, heparin cofactor II and tissue factor pathway inhibitor (TFPI) (145–147), resulting in a pro-coagulant microenvironment, and NET-associated oxidative enzymes oxidize high density lipoprotein making it proatherogenic (57). Moreover, SLE serum induces neutrophil autophagy and NETosis by upregulating expression of hypoxia-response and stress response protein REDD1 (148). Through this pathway NETs are decorated with tissue factor and IL-17A, making them highly prothrombotic.

However, there are controversies regarding NETs and their role in autoimmune diseases (120). Independent studies have demonstrated that SLE mouse models with knocked-out or inhibited NOX2 or PAD4, thus with deficient NET formation, display either a more severe or unchanged disease phenotype compared to mice with normal NET releasing ability (89, 90, 149, 150). In the case of NOX2 pathway blockade, this effect is probably dependent on the many effects of NOX2-derived ROS as regulators of inflammation, as discussed previously in this review. Potential protective effects of NETs include aggregation and degradation of inflammatory cytokines via NET-related serine proteases (151).

### Neutrophil Interactions With Other Cells of the Immune System

Neutrophils cross-talk with most cells of the immune system, reviewed in Scapini and Cassatella (152). As previously discussed in this article, neutrophils produce large amounts of ROS during oxidative burst, which has several immunoregulatory effects on other cells of the immune system.

Neutrophils produce several different cytokines, both pro- and anti-inflammatory, as well as immunomodulatory and chemotactic cytokines (153). The cytokine profile of patients with both SLE and APS differ from that of healthy individuals, with increased levels of many pro-inflammatory mediators (60, 154), which might be a consequence of the many neutrophil abnormalities seen in SLE as discussed in this review. IFN-α, a key cytokine involved in many aspects of both SLE and APS can be produced in neutrophils (24). The amount of IFN produced by a neutrophil is only a fraction of what can be produced by a pDC but considering the vast amount of neutrophils present in the circulation the neutrophil is probably a more important source of IFN than previously considered. IFN produced by neutrophils in the bone marrow in SLE has been demonstrated to disturb B-cell development (155). Neutrophils also produce B-cell activating factor (BAFF/BLyS) which promotes activation of autoreactive B-cells and antibody production in SLE (156, 157). T-cells represent a key checkpoint for autoreactive B-cells and BAFF affects classic T-B cell interactions. Activated CD4+ cells migrate to B-cell follicles and transforming into follicular T-cells, which promotes formation of germinal centers and sustains the activation of B-cells and eventually long-lived plasma cells (158). Neutrophils also possesses the capacity of antigen presentation (159, 160). Antigen presentation by professional antigen presenting cells activates T-cells producing e.g., IFN-γ. It has been shown that these cytokines stimulate upregulation of MHC II and costimulatory molecules on neutrophils, enabling them to present antigens to T-cells, and an upregulation of MHC II on neutrophils is found in rheumatoid arthritis and systemic small vessel vasculitis (161, 162). Thus, neutrophils may also play a role in the pathogenesis of autoimmune diseases by presenting autoantigens.

Neutrophils also interact with other immune cells via direct cell-cell contact. Especially platelets communicate with neutrophils in this manner (163). Increased platelet-neutrophil interaction has been detected in SLE and activated platelets binding to neutrophils facilitates NET release (164, 165), suggesting that neutrophils and platelets act together in this process in SLE (166). Neutrophils are also dependent on platelets during migration, as clusters of P-selectin glycoprotein ligand 1 (PSGL-1) needs to bind P-selectin on activated platelets in order to express receptors driving neutrophil migration (167). Blockade of PSGL-1 mediated neutrophil-platelet interactions results in decreased neutrophil migration and protection against thrombo-inflammatory injury. PSGL-1 is upregulated in APS patient neutrophils and PSGL-1 deficient mice are protected from antiphospholipid antibody mediated thrombosis (37). Activated platelets release their granules with proinflammatory chemokines and cytokines capable of promoting chemotaxis of neutrophils (168). Neutrophil-platelet interactions are important in thromboinflammation and probably involved in the cardiovascular manifestations in SLE and APS by mechanisms mentioned earlier; generation of NETs and release of neutrophil constituents such as elastase, cathepsin G, and tissue factor. However, the communication with platelets may also contribute to resolution of inflammation. Platelets potentiate intra- and extracellular generation of ROS and myeloperoxidase from stimulated neutrophils and enhance Fcγ receptor-mediated phagocytosis (169).

## Concluding Remarks

Neutrophils have a limited amount of effector functions, but an imbalance in any of them will have large effects on the whole immune system. SLE and APS are two autoimmune diseases that can occur together or separately in a patient with overlapping pathogenesis and may be considered as a continuum with a sliding scale of clinical manifestations. Patients suffering from SLE or APS have an increased cardiovascular morbidity compared to the healthy population, which to a significant extent is related to abnormal neutrophil function. Autoimmunity is generally thought of as a phenomenon mainly dependent on activation of an autoreactive adaptive immune system, but in SLE and APS a functional neutrophil is important for preventing loss of tolerance via immune regulation and clearance.

## Author Contributions

LW and AB contributed to the original idea and manuscript writing. SA and PL participated in the planning and writing of sections of the manuscript.

### Conflict of Interest

The authors declare that the research was conducted in the absence of any commercial or financial relationships that could be construed as a potential conflict of interest.
